# Prognostic value of various subtypes of extracellular DNA in ovarian cancer patients

**DOI:** 10.1186/s13048-018-0459-z

**Published:** 2018-09-22

**Authors:** Katarina Kalavska, Tomas Minarik, Barbora Vlkova, Denisa Manasova, Michaela Kubickova, Andrej Jurik, Jozef Mardiak, Jozef Sufliarsky, Peter Celec, Michal Mego

**Affiliations:** 1 2nd Department of Oncology, Faculty of Medicine, Comenius University and National Cancer Institute, Klenova 1, 833 10 Bratislava, Slovak Republic; 2 Translational Research Unit, Faculty of Medicine, Comenius University, Bratislava, Slovakia; 3 National Cancer Institute, Bratislava, Slovakia; 4 Institute of Molecular Biomedicine, Faculty of Medicine, Comenius University, Bratislava, Slovakia

**Keywords:** Extracellular DNA, Plasma DNA, Ovarian cancer, Prognostic marker

## Abstract

**Background:**

Patients with ovarian cancer represent a heterogeneous population with a variable prognosis and response to chemotherapy. Plasma DNA has been shown to have a prognostic value in different types of cancer including ovarian carcinoma. Whether total circulating DNA, which can be assessed much easier without knowing the tumor-specific mutations, has similar informative value is currently unknown. The aim of this study was to evaluate the prognostic value of extracellular DNA in advanced ovarian cancer.

**Methods:**

This prospective study included 67 patients (pts) with ovarian cancer treated with 1st line paclitaxel and carboplatin (25 pts) and paclitaxel, carboplatin and bevacizumab (42 pts). Thirty-five patients had optimal surgical debulking before chemotherapy. Extracellular DNA was quantified using real time PCR before administration of chemotherapy (67 pts) and after 6 cycles of chemotherapy (44 pts).

**Results:**

Total extracellular DNA (ecDNA), as well as extracellular DNA of nuclear (nDNA) and mitochondrial origin (mtDNA) significantly (*p* < 0.05) decreased after 6 cycles of chemotherapy (by 54%, 63% and 52%, respectively. Patients with stage I disease had significantly lower mtDNA compared to patients with stage II-IV (8604 vs. 16, 984 ge/mL, *p* = 0.03). Patients with lower baseline nDNA had superior progression-free (HR = 0.35 (0.14–0.86)) and overall survival (HR = 0.18 (0.04–0.77). The prognostic value of nDNA was confirmed independent of tumor stage and confirmed in multivariate analysis.

**Conclusions:**

Our data suggest that ecDNA of both, nuclear and mitochondrial origin could be added to prognostic markers in ovarian cancer. Analysis of ecDNA does not require the knowledge of tumor-specific mutations in contrast to the quantification of tumor-derived ecDNA. Study of the dynamics and cell type-specific source of the ecDNA could shed light on its biology in cancer and might help to direct the treatment of ovarian cancer.

## Background

Ovarian cancer represents the most lethal gynecologic malignancy and the fifth most common cause of cancer death in woman [1]. The high mortality of this disease may be due to the lack of early diagnostic and screening methods. Most ovarian cancer patients are diagnosed at an advanced stage of the disease (FIGO Stage III and IV) characterized by widely metastatic disease with 10-year survival rate 5–21% [2]. In contrast, survival rates for patients diagnosed at early stage I (tumor confined to the ovary) are nearly 90% [3]. Thus, patients with ovarian cancer may serve as a heterogeneous population with a variable prognosis and response to chemotherapy. Cancer antigen CA-125 represent frequently used biomarker for predicting prognosis and therapeutic response in ovarian cancer patients. Its levels together with imaging techniques are widely used in patient management. However, sensitivity of current imaging techniques is limited. Therefore, there is an urgent need for development of novel biomarkers effectively predicting therapeutic response and prognosis of patients with ovarian cancer [4–6]. Recently, several studies are focused on quantitative analysis of extracellular DNA as on novel non-invasive approach to follow patients after treatment, monitor therapeutic response and promote earlier detection of recurrences in different types of cancer including ovarian carcinoma [7, 8].

Extracellular DNA include double-stranded molecules in the form of short fragments (between 70 and 200 bp) or long fragments up to 21 kb accessible for analysis from plasma or serum of cancer patients [9, 10]. The presence of extracellular DNA in the circulation is explained by releasing of nuclear and mitochondrial DNA by both normal and tumor cells into the circulation through the destruction of apoptic and necrotic cells [9, 11].

Extracellular DNA as a liquid biopsy can also serve as valuable tool revealing tumor genetic changes, including the acquisition of resistance associated mutations during therapy. Various types of DNA alterations (point mutations, microsatellite instabilities, DNA hypermethylations and loses of heterozygosity), identical to the ones detected in the primary tumor tissue, were detected in extracellular DNA [12–14]. The study Gray et al. analyzing extracellular DNA in metastatic melanoma patients showed that lower extracellular DNA levels prior to treatment significantly correlated with response to therapy and prolonged progression free survival, regardless to therapy type. Moreover, detection of circulating mutant *NRAS* preceded radiological detection of disease progression [12]. Similarly, quantitative analysis of tumor associated mutant *BRAF* extracellular DNA revealed that higher overall response rate to BRAF inhibitors and longer progression free survival were seen in melanoma patients with lower concentration of basal mutant *BRAF* extracellular DNA [15, 16]. Tsao et al. also showed correlation between changes in extracellular DNA levels in melanoma patients and their disease status [17]. Fiegl et al. measured *RASSF1A* DNA methylation of extracellular DNA in the serum to monitor response of women with breast cancer and presence of *RASSF1A* methylation 1 year after primary surgery was evaluated as an independent predictor of poor outcome in these patients [18].

Due to significant differences in extracellular DNA levels between ovarian cancer patients before and after chemotherapy, we may suppose that the quantification of plasma DNA might serve as a new method to monitor effect of chemotherapy [19, 20]. Moreover, the extracellular DNA levels also correlate with time of recurrence in these patients [19]. The study Steffensen et al. evaluating 144 patients with epithelial ovarian carcinoma treated with bevacizumab showed significant association between high levels of extracellular DNA and shorter progression free survival and overall survival [21]. In addition, the treatment efficacy in ovarian carcinoma may be also monitored by detection of mutations in extracellular DNA. It was showed that 12 of 27 ovarian carcinoma patients had mutations of p53 in the cancer tissue. In 2 of those 12 cases identical mutations in the DNA of their preoperative plasma were detected. Interestingly, mutant DNA was undetected in after surgery follow-up of these two patients with p53 mutations in their extracellular DNA; however, in one case, the p53 mutation resurfaced 16 months after surgery [22]. Moreover, the predictive value of extracellular DNA was also revealed by studies showing that very high pre-operative plasma levels of extracellular *RAB25* were significantly associated with decreased patients’ survival. Therefore, plasma levels of cfDNA may represent an independent predictor of death from ovarian cancer [4, 23].

Kamat et al. used an orthotopic ovarian cancer mouse model to determine the relationship between the kinetics of tumor-specific extracellular DNA and tumor growth. They found that extracellular DNA levels closely correlated with tumor burden and varied during treatment with two separate therapeutic regimens (cytotoxic chemotherapy and anti-angiogenic treatment with monoclonal antibodies). Specifically, the extracellular DNA levels rapidly declined after an initial rise. The peak in ecDNA levels was associated with an increase in the rate of apoptosis in treated ovarian tumors [11].

The higher abundance and shorter length of mitochondrial genome (mtDNA) as opposed to nuclear genome allow the suggestion that the amount of circulating extracellular mtDNA should be higher than extracellular nuclear DNA. Thus quantification of the extracellular mtDNA may increase sensitivity of this method [24, 25]. Moreover, presence of mtDNA in circulation may inform on cellular stress and cellular senescence [26]. The study Zachariah et al. showed that levels of extracellular mtDNA and nuclear DNA in plasma of patients with epithelial ovarian cancer were significantly elevated in comparison to healthy controls amd patients with benign ovarian lesions. However, no correlation between extracellular mtDNA a nuclear DNA was found [24].

Whether total circulating DNA, which can be assessed much easier without knowing the tumor-specific mutations, has similar informative value is currently unknown. The aim of this study was to evaluate the prognostic value of the quantity of extracellular DNA in advanced ovarian cancer.

## Results

### Patients’ characteristics

Patients’ characteristics are shown in Table 1**.** This prospective study included 67 patients with ovarian cancer treated with 1st line paclitaxel and carboplatin (25 pts) and paclitaxel, carboplatin and bevacizumab (42 pts). Thirty-five patients had optimal surgical debulking before chemotherapy (less than 1 cm residual disease). Thirty three tumors with serous histology in our study were grade 3. Six patients with serous histology had stage I disease (grade 2 had 1 patient, grade 3 had 3 patients and 2 patients had unknown grading). Six patients had endometrioid tumors (three grade 3, two grade 2, one patient had unknown grading), one of them had stage I, one stage II and 4 had stage III, 4 patients had clear cell carcinoma, all grade III, 3 of them had stage I and the last had stage III. Three patients had mucinous carcinoma, two had stage I and one had stage III disease (1 patient grade 1, 2 patients had unknown grading). One patient had undifferentiated carcinoma, grade III, stage III.

**Table 1:** Patients’ characteristics

	N	%	Total extracellular DNA ng/mL median	*p*-value	Extracellular nuclear DNA ge/mL median	*p*-value	Extracellular mitochondrial DNA ge/mL median	*p*-value
All patients	67	100.0	8.3		2266		16,526	NA
Surgical Debulking								
Optimal	35	52.2	8.26	0.35	1944	0.13	11,422	0.09
Suboptimal	32	47.8	8.94		2584		20,297	
Histology								
Serous	53	79.1	9	0.31	2422	0.63	16,526	0.70
Other	14	20.9	6.16		1919		13,117	
Grade								
1 and 2	14	20.9	7.63	0.47	2083	0.49	16,470	0.90
3	41	61.2	8.32		2266		15,646	
Unknown	12	17.9						
Stage								
1	12	17.9	5.3	0.58	1862	0.45	8604	0.16
2	7	10.4	8.26		2422		16,526	
3	43	64.2	9		2455		17,575	
4	5	7.5	9.7		1736		16,413	
Stage								
1	12	17.9	5.3	0.19	1862	0.18	8604	0.03
2 to 4	55	82.1	9		2438		16,984	
		0.0						
Chemotherapy								
paclitaxel+carboplatin	25	37.3	6.26	0.21	1893	0.19	14,314	0.76
paclitaxel+carboplatin+bevacizumab	42	62.7	9.17		2447		16,755	

Extracellular plasma DNA was quantified using real time PCR before administration of chemotherapy (67 pts) and after 6 cycles of chemotherapy (44 pts).

### Association between cfDNA and patients/tumor characteristics

Total extracellular DNA (ecDNA), as well as extracellular DNA of nuclear (nDNA) and mitochondrial origin (mtDNA) significantly (*p* < 0.05) decreased after 6 cycles of chemotherapy (by 54%, 63% and 52%, respectively) (Table 2). Discrepancy between number of samples before treatment (*n* = 67) and after the 6 cycles of chemotherapy (*N* = 44) was due to logistic reason and was not related to disease/patient’s characteristic, only 3 of 67 patients experienced disease progression before 6 cycles of chemotherapy. Patients with stage I disease had significantly lower mtDNA compared to patients with stage II-IV (8604 vs. 16, 984 ge/mL, *p* = 0.03). Pretreatment level of all cfDNA subtypes were significantly higher at baseline before chemotherapy compared to levels after 6 cycles of chemotherapy (Table 1).

**Table 2:** Comparison between pre-treatment and matched post-treatment extracellular DNA (*N* = 44)

	Pre-treatmnet (median)	After-treatment (median)	*p*-value
Total extracellularDNA (ng/mL)	7.4	3.4	0.002
Extracellular nuclear DNA (ge/mL)	2163.5	791.0	0.004
Extracellular mitochondrial DNA (ge/mL)	12,304.0	5915.5	0.001

### Prognostic value of cfDNA

Median follow-up for all patients was 19.9 months (range: 0.2–34.2 months). Patients with lower baseline nDNA had superior progression-free (HR = 0.35 (0.14–0.86)) and overall survival (HR = 0.18 (0.04–0.77) (Table 3), while baseline total ecDNA and mtDNA were not prognostic for PFS nor for OS. In opposite patients with higher posttreatment mtDNA, but not total and nDNA, had inferior PFS compared to patients with lower posttreatment mtDNA (Table 4).

**Table 3:** Prognostic value of pre-treatment extracellular DNA

Variable	PFS			OS		
	HR	95% CI	*p*-value	HR	95% CI	*p*-value
Total extracelular DNA	0.62	0.25–1.53	0.28	0.27	0.06–1.24	0.09
Extracellular nuclear DNA	0.35	0.14–0.86	0.02	0.18	0.04–0.77	0.06
Extracellular mitochondrial DNA	0.83	0.34–2.00	0.67	0.57	0.13–2.55	0.45

**Table 4:** Prognostic value of post-treatment extracellular DNA

Variable	PFS			OS		
	HR	95% CI	*p*-value	HR	95% CI	*p*-value
Total extracellular DNA(ng/mL)	1.23	0.41–3.68	0.72	0.88	0.14–5.4	0.89
Extracellular nuclear DNA (ge/mL)	0.7	0.23–2.16	0.51	0.97	0.16–5.83	0.97
Extracellular mitochondrial DNA (ge/mL)	3.45	1.16–10.24	0.04	0.41	0.07–2.49	0.27

In multivariate analysis, the prognostic value of baseline nDNA was confirmed independent of tumor stage and debulking status (Table 5) while posttreatment mtDNA were not independent from tumor stage (data not shown).

**Table 5:** Multivariate analysis of factors associated with progression free survival

Variable	Progression-free survival (HR (95% C.I.), *P* – value)
Stage	
Stage IV vs. stage I	5.20 (0.44–61.89), *P* = 0.19
Stage III vs. stage I	2.18 (0.22–21.45), *P* = 0.50
Stage II vs. stage I	1.34 (0.08–22.94), *P* = 0.84
Debulking	
Suboptimal vs. optimal	1.79 (0.57–5.62). *P* = 0.32
Pre-treatment extracellular nuclear DNA (ng/mL)	
High vs. Low	3.11 (1.03–9.45). *P* = 0.045

## Discussion

The presence of cfDNA within the plasma was initially reported by Mandel and Metais in 1948 [27, 28]. Currently there is a great interest in the potential use of cfDNA in many different areas of biomedical research, including cancer biomarker research [4, 29, 30]. Interestingly, cfDNA may serve as a surrogate marker in monitoring of NETs (neutrophil extracellular traps) playing role as procoagulant and prothrombotic factor in thrombosis. The study of Demers et al. revealed that malignant neutrophils are more prone to NET formation. This elevated sensitivity to NET generation suggests that malignant process through a systemic effect on the host can lead to increased NET formation and cancer-associated thrombosis [31]. Several studies have demonstrated that cancer patients have generally higher levels of total cfDNA (from both normal and malignant cells) compared to healthy individuals [23, 31, 32]. However, it is not yet clear whether the majority of cfDNA present in plasma of cancer patients is tumor-derived [33, 34]. The proportion of cfDNA originated from tumor tissue is not only associated by the state and size of the tumor [35], but also by clearance, degradation, lymphatic circulation, and other physiological blood processing tumor [6, 36, 37] Circulating tumor DNA (ctDNA) is characterized by specific genetic alterations such as methylation or mutation present in DNA of malignant cells [8]. Many studies have evaluated prognostic value of both cfDNA and ctDNA and investigated their role as a marker of response to therapy [13, 14, 19, 20]. Although reported data are abundant, results differ among studies. To the best our knowledge, only two meta-analyses have shown the diagnostic accuracy of quantitative analysis of cfDNA is at least the same as the conventional biomarkers for diagnosis of lung cancer [29, 38] and hepatocellular carcinoma [29, 36]. Moreover, meta-analysis published by Ocana et al. showed correlation between the high levels of cfDNA and the worse survival in solid tumors [37]. Screening biomarkers potentially used in diagnosis of ovarian cancer have been widely studied, but only few have satisfactory specificity and sensitivity for clinical applications [4]. Therefore, improving of early detection methods and finding of novel diagnostic biomarkers for ovarian cancer is highly warranted.

In present translational study, we sought to define the prognostic value of various subtypes of cfDNA in ovarian cancer patients. The total ecDNA, as well as DNA and mtDNA was significantly decreased after 6 cycles of chemotherapy (by 54%, 63% and 52%, respectively). We also found that ovarian cancer patients with stage I disease had significantly lower mtDNA compared to patients with stage II-IV. Moreover, patients with lower baseline nDNA had superior progression-free survival. Patients with optimal debulking had better PFS and OS compared to patients with suboptimal debulking, interestingly, statistically significant difference in pre-chemotherapy extracellular DNA was not detected between optimally debulked and suboptimally debulked patients. In multivariate analysis was the prognostic value of nDNA confirmed independent of tumor stage and tumor debulking.

Recently, the quantitative analysis of circulating cf. DNA in ovarian cancer patients has attracted increasing attention. The meta-analysis published by Zhou et al. showed that quantitative analysis of cfDNA has poor sensitivity (0.70 (95% CI, 0.65–0.74) but acceptable specificity (0.90 (95% CI, 0.87–0.93) for diagnosis of ovarian cancer [29]. Data obtained in our study indicate that ecDNA of both, nuclear and mitochondrial origin could be added to prognostic markers in ovarian cancer. ecDNA as a prognostic marker has currently been evaluated in several solid tumors [39–42]. In patients with non-small cell lung cancer was found significant correlation between tumor progression and increasing plasma DNA [43]. Wei and colleagues reported the association between surgical resection of the tumor and significant decline in the Epstein-Barr virus (EBV) DNA copy numbers in nasopharyngeal carcinoma patients [40]. Moreover, significantly elevated pre-therapy plasma EBV DNA levels were also detected as a predictor of clinical outcome in nasopharyngeal carcinoma patients with early stage of disease [42]. The study Kamat et al. reported that preoperative levels of cfDNA were significantly increased in ovarian cancer patients compared to patients with benign ovarian disease and healthy controls. Moreover, high levels of ecDNA were associated with high-grade and high-stage of disease [4]. These data are consistent to our results, where ovarian cancer patients with stage I disease had significantly lower mtDNA when compared to patients with stage II-IV. Patients with lower baseline nDNA had superior progression-free and overall survival. On the other hand, although the study Zachariah et al. have reported increased levels of both ecDNA and mtDNA in patients with ovarian cancer compared to healthy controls, the association between ecDNA and prognosis was not confirmed [24]. Disparity between the extracellular mt DNA, nDNA and total DNA may be explained by different release mechanisms in the events of cell turnover in circulation [24]. In addition, our study also showed significant decline of ecDNA, as well as nDNA and mtDNA after 6 cycles of chemotherapy. Similar results were seen in study Kamat et al. using an orthotopic mouse model of ovarian cancer. In this study they have demonstrated that tumor-specific cfDNA correlated with tumor burden and levels decreased in response to chemotherapy [11]. Another study analyzing 144 patients with epithelial ovarian cancer treated with bevacizumab showed significantly shorted PFS and OS in patients with high levels of cfDNA in the blood [21]. Interestingly, it was showed that absolute amounts of cfDNA analyzed in cancer patients vary between different studied and whether the DNA is isolated by plasma-based or serum-based assays. cfDNA derived from plasma samples showed higher level of sensitivity but lower level of specificity. While Zhou et al. concluded that the best source for reliable cfDNA detection cannot be determined by current evidence; Thijssen et al. supposed that cfDNA isolated from plasma samples better reflects the in vivo levels of cfDNA [29, 44]. Furthermore ROC curves of cfDNA presented by Shao et al. revealed that the sensitivity and specificity of serum cfDNA levels were 88.9 and 89.5%, while cfDNA combined with CA125 and HE-4 impoved sensitivity up to 91.67% [45]. Storage of cfDNA (EDTA or citrate) and centrifugation speed by isolation of cfDNA may also affect detection levels of cfDNA [41, 46]. These factors may also represent the limitations of our study. In addition, our study did not compare the diagnostic value of cfDNA with other diagnostic markers (eg. CA125), thus could not compare its diagnostic accurancy with clinically used diagnostic markers for ovarian cancer.

## Conclusions

In conclusion, our data suggest that ecDNA of both, nuclear and mitochondrial origin could be added to prognostic markers in ovarian cancer, however further studies with larger sample size are needed to define their role in the context of existing prognostic markers. Analysis of ecDNA does not require the knowledge of tumor-specific mutations in contrast to the quantification of tumor-derived ecDNA. Study of the dynamics and cell type-specific source of the ecDNA could shed light on its biology in cancer and might help to direct the treatment of ovarian cancer.

## Methods

### Patients

Into this translational study were included 67 patients with ovarian cancer treated with first line chemotherapy from February 2014 to March 2016 in the National Cancer Institute in Slovakia. Patients with concurrent malignancy other than non-melanoma skin cancer in the previous 5 years were excluded. All patients were chemo-naïve. Median time between last surgery and blood draw for analysis was 20 days (range: 7–108 days). All patients were treated with primary cytoreductive surgery, no patients with neoadjuvant chemotherapy were included. In all patients, data regarding age, tumor histology, clinical stage, type/number of metastatic sites and delivery of systemic therapy were recorded and compared with extracellular DNA levels. The study was approved by the Institutional Review Board (IRB) of the National Cancer Institute of Slovakia. All patients enrolled into this study gave informed consent.

### Plasma isolation

Total venous peripheral blood volume (12 mL) was collected in EDTA-treated tubes and centrifuged at 1000 g for 10 min at room temperature within 2 h of venipuncture. To avoid cellular contamination, plasma was carefully harvested and centrifuged again at 1000 g for 10 min at room temperature. The cell-free plasma samples were cryopreserved at − 80 °C until further analyses.

### Circulating plasma DNA measurement

Plasma samples were re-centrifuged at 16000×g and 4 °C to pellet the cellular debris. The final supernatant (200 μl) was used for plasma ecDNA isolation (QIAamp DNA Blood Mini Kit, Qiagen, Hilden, Germany). The concentration of total ecDNA was measured fluorometrically using Qubit 2.0 Fluorometer and the dsDNA HS Assay kit (Thermo Fisher Scientific, Wilmington, DE, USA). Real time PCR targeting the nuclear beta globin gene and the mitochondrial cytochrome b gene was used for the quantification of ecDNA of nuclear and mitochondrial origin as described previously [31]. The QuantiTect SYBR Green PCR Kit (Qiagen, Hilden, Germany) and the MasterCycler RealPlex cycler (Eppendorf, Hamburg, Germany) were used the real time PCR analysis. The efficiency of both reactions was between 90 and 110% and the resulting Ct values were used for the calculation of genome equivalents (GE) per ml of plasma. All PCR products were checked using the melting curve analysis to ensure a single product was amplified.

### Statistical analysis

The characteristics of the cohort were summarized using the median (range) for continuous variables and frequency (percentage) for categorical variables. Normality of distribution was tested using the Kolmogorov-Smirnoff test. For normally distributed data, differences between the groups were tested using the Student *t*-test or analysis of variance (ANOVA) with corrections according to Bonferroni or Tamhane depending on the homogeneity of variance. The nonparametric Mann-Whitney *U* test was used for non-normally distributed data. Pearson or Spearman correlations test was used according to the normality of data. Categorical data were tested using the Fisher’s exact test or Chi square test.

Median follow-up period was calculated as a median observation time among all patients and among those still alive at the time of their last follow-up. Progression-free survival (PFS) was calculated from the date of the starting treatment with chemotherapy to the date of progression or death or the date of the last adequate follow-up. Overall survival (OS) was calculated from the date of starting treatment with chemotherapy to the date of death or last follow-up. Survival rates were estimated using the Kaplan-Meier product limit method and were compared with the log-rank test to determine significance. cfDNA level data were dichotomized into high and low groups based on the cfDNA level median value of all samples. A multivariate Cox proportional hazards model for PFS and OS was used to assess differences in outcome on the basis of cfDNA level, debulking status and stage of disease. All *p*-values presented are two-sided and were considered significant if less than 0.05. Statistical analyses were performed using NCSS 2007 software (Hintze J, 2007, Kaysville, UT, USA).
